# Sorption of Aromatic Compounds with Copolymer Sorbent Materials Containing β-Cyclodextrin

**DOI:** 10.3390/ma4091528

**Published:** 2011-08-29

**Authors:** Lee D. Wilson, Mohamed H. Mohamed, Christopher L. Berhaut

**Affiliations:** 1 Department of Chemistry, 110 Science Place (Rm. 156), University of Saskatchewan, Saskatoon, SK S7N 5C9, Canada; 2 Aquatic Ecosystems Protection Research Division, Water Science and Technology Directorate, 11 Innovation Boulevard, Saskatoon, SK S7N 3H5, Canada; E-Mail: mom133@mail.usask.ca; 3 École Nationale Superieure de Chimie de Montpellier, 8 Rue de l’Ecole Normale, Montpellier 34296 CEDEX 5, France; E-Mail: christopher.logan@wanadoo.fr

**Keywords:** β-cyclodextrin, sorption, dichlorophenol, isotherm, pentachorophenol, 2,4-dichlorophenoxy acetic acid, urethane copolymer

## Abstract

Urethane copolymer sorbent materials that incorporate β-cyclodextrin (CD) have been prepared and their sorption properties with chlorinated aromatic compounds (*i.e*., pentachlorophenol, 2,4-dichlorophenol and 2,4-dichlorophenoxy acetic acid) have been evaluated. The sorption properties of granular activated carbon (GAC) were similarly compared in aqueous solution at variable pH conditions. The sorbents displayed variable BET surface areas as follows: MDI-X copolymers (< 10^1^ m^2^/g), CDI-X copolymers (< 10^1^ m^2^/g), and granular activated carbon (GAC ~10^3^ m^2^/g). The sorption capacities for the copolymers sorbents are listed in descending order, as follows: GAC > CDI-3 copolymer ≈ MDI-3 copolymer. The sorption capacity for the aromatic adsorbates with each sorbent are listed in descending order, as follows: 2,4-dichlorophenol > 2,4-dichlorophenoxy acetic acid > pentachlorophenol. In general, the differences in the sorption properties of the copolymer sorbents with the chlorinated organics were related to the following factors: (*i*) surface area of the sorbent; (*ii*) CD content and accessibility; and (*iii*) and the chemical nature of the sorbent material.

## 1. Introduction

**Scheme 1:**
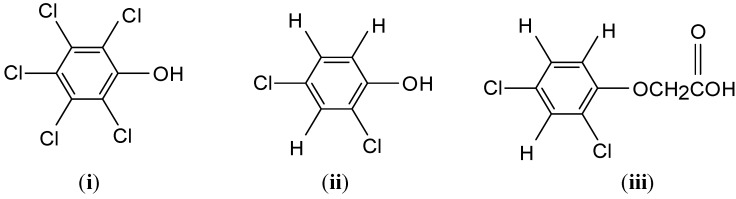
Chemical structures of chlorinated aromatic adsorbates investigated in this work: (**i**) pentachlorophenol (PCP); (**ii**) 2,4-dichlorophenol (2,4-DCP), and (**iii**) 2,4-dichlorophenoxy acetic acid (2,4-D).

Phenolic compounds are ubiquitous in nature and are widely used as raw materials in the chemical industry [1] and other applications including petrochemical processing, kraft pulp and paper production, and olive oil products [1,2]. The buildup and transport of chlorophenol-based pollutants (e.g., biocides, petrochemicals, and pharmaceuticals) represent an important environmental issue which may adversely impact aquatic ecosystems, biodiversity, and human health [3,4,5,6,7,8]. One of the eight millennium goals of the United Nations Environment Program (UNEP) is the provision of clean drinking water and its mandate is to reduce the proportion of people without sustainable access to safe drinking water by half in 2015 [9]. Therefore, there is an urgent need to develop novel materials and innovative methods to address the removal of phenols from contaminated aquatic environments. Chlorophenol and its conjugates have been used as wood preservatives and biocides despite their acute toxicity and widespread occurrence [3,4,5,6,7,8]. For example; 2,4-dichlorophenol (2,4-DCP) is a toxic organic compound that has been widely used as an agrochemical and as a preservative for wood, leather and glue. Scheme 1 illustrates the molecular structure of a series of model chlorinated aromatic compounds that represent significant concern for human health and the environment.

**Scheme 2:**
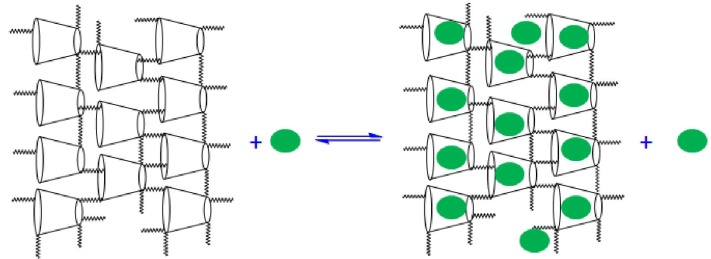
Inclusion and interfacial equilibrium binding modes in CD-based copolymer sorbent materials. The tori represent β-CD and the line segments represent the crosslinker domains of the copolymer framework. The green ovals represent an adsorbate guest molecule.

Advances in polymer chemistry have afforded the development of novel synthetically engineered sorbent materials that incorporate carbohydrate molecules such as cyclodextrins (CDs) within a polymeric framework [10,11,12,13,14,15,16,17,18,19,20,21] CDs are water soluble cyclic oligosaccharides composed of six, seven, or eight glucopyranose units linked by α-1,4-glycosidic bonds; hereafter, they are referred to as α-, β-, and γ-cyclodextrin. β-CD is of particular interest owing to it size (depth of 7.8 Å and inner average diameter of 7.8 Å), unique physicochemical properties, and host-guest chemistry [10,16,22] The torus-shaped macrocycle structure of β-CD has a hydrophilic exterior and a lipophilic interior which is suitably sized for the inclusion of benzyl and naphthyl moieties [23]. CDs were recently used as potential pore templates to form microporous and nanoporous materials because of their well-defined structure, relatively low toxicity, versatility, and the ability to form inclusion and interfacial complexes [24,25], illustrated in Scheme 2. The sequestration of chlorophenols from contaminated aquatic environments and soils have been investigated using naturally occurring and synthetically modified materials [7,15,26]. Recent studies by Mohamed *et al.* indicate that synthetically engineered copolymers containing β-CD represent a versatile class of sorbent materials for solid-solution adsorption processes [13,14]. In particular, CD-based sorbent materials are of great interest because of their tunable physicochemical and sorption properties. Selective adsorption of adsorbates according to their size and physical properties were observed for phenolate and naphthenate anions in aqueous solution [27]. Thus, β-CD copolymer materials have significant potential for the sequestration of chlorophenol and related organic contaminants in aquatic and soil media to safe concentration levels [15,17,18,28].

The objectives of this study were to investigate the solid-solution isotherm properties of two structurally related (*i.e.*, aliphatic *vs*. aromatic) β-CD urethane copolymers with a series of chlorinated aromatic adsorbates (*cf*. Scheme 1) and *p*-nitrophenol (PNP) in aqueous solution. The copolymer solid-solution sorption properties are compared with granular activated carbon (GAC) in aqueous solution at alkaline pH and 295 K. The results of this sorption study will to contribute to a better understanding of the sorption behavior of copolymer materials toward chlorinated aromatics and their potential utility in aquatic and soil environments. The development of novel sorbents of this type may offer potential large-scale methods (e.g., fluidized bed reactors) to sequester recalcitrant and toxic environmental contaminants [28,29,30,31,32].

## 2. Methods

### 2.1. Materials

Granular Activated Carbon (GAC; Norit Rox 0.8) was obtained from Norit America and used as received. GAC materials were ground and sieved through a size 40 mesh (≤ 0.42 mm) particle sieve and subsequently dried under vacuum. The following diisocyanate crosslinkers were used in this work: methylenediphenyl diisocyanate (MDI; Aldrich), and dicyclohexylmethane-4,4’-disiisocyante (CDI; Aldrich) [33]. β-cyclodextrin (β-CD), pentachlorophenol (PCP), 2,4-dichlorophenol (2,4-DCP), 2,4-dichlorophenoxy acetic acid (2,4-D), and *p*-nitrophenol (PNP) were obtained from Aldrich and used as received without any further purification. The synthesis of the urethane copolymers containing β-CD was adopted from a published procedure [33].

Solid state ^13^C CP-MAS NMR spectra, IR spectra, TGA, elemental analysis (Perkin Elmer 2400 CHN Elemental Analyzer) were obtained and reported elsewhere [13,27,33]. Porosimetry (Micromeritics ASAP 2010) was used to estimate the surface area and pore structure characteristics of the sorbent materials [34].

### 2.2. Sorption Methods

A range of concentrations of adsorbate (0.1–8 mM) were prepared at pH 9.00 in 0.01 M NaHCO_3_ buffer. The molar absorptivity of the adsorbates was determined using the Beer-Lambert law at the same conditions described for sorption experiments. The experimental values are in good agreement with previous reports [28]. To a 10 mL glass bottle with Teflon sure seal^®^cap liners, constant amounts of solid polymer (~20 mg) were added to a fixed volume (7.00 mL) of an adsorbate solution. The vials were further sealed with parafilm between the cap and glass bottle and were placed at room temperature in a horizontal shaker (Heto Birkeroder Type: TBSH02, Denmark) at 1500 rpm to equilibrate for 24 h. The equilibrium concentrations of adsorbate were determined using a double beam spectrophotometer (Varian CARY 100) at room temperature (295 ± 0.5 K) by monitoring the absorbance changes at the λ_max_. The molar absorptivities were determined as follows; 3178 ± 159 M^−1^ cm^−1^ (DCP), 4501 ± 225 M^−1^ cm^−1^ (PCP), 1483 ± 74.2 M^−1^ cm^−1^ (2,4-D), and 9287 ± 464 M^−1^ cm^−1^ (PNP), respectively. An average of 15–20 data points were obtained for each sorption isotherm. For analyses of residual adsorbate concentration in solution, absorbance values were recorded for each sample at the respective λ_max_ for each aromatic adsorbate, respectively. The dye-based method was adapted from a previous study which utilized PNP as the adsorbate probe [13].

### 2.3. Data Analysis

The solution sorption results are plots of equilibrium concentration of adsorbate in the copolymer phase (Q_e_) removed from solution according to the mass of sorbate *versus* the residual equilibrium concentration of adsorbate (C_e_) in aqueous solution. Q_e_ is defined by Equation 1, where C_o_ is the initial adsorbate concentration, V is the volume of solution, and m is the mass of sorbent.

(1) $$Q_{e}=\frac{(C_{o}-C_{e})\times V}{m}$$

The Sips isotherm model [35] was used to analyze the experimental equilibrium sorption data. This model represents a generalized isotherm model which can be interpreted in the context of monolayer or multilayer adsorption processes, in accordance with the adjustable exponent parameter (n_s_; *cf.* Equation 2). The Sips models accounts for Langmuir- and Freundlich-type behavior at certain limiting conditions. Langmuir isotherm behavior is predicted when n_s_ = 1; whereas, Freundlich behavior is predicted when ${\left(K_{s}C_{e}\right)}^{n_{s}}$<<< 1. The sorbent-adsorbate binding affinity can be related to the equilibrium constants (K_S_) appearing in Equation (2). The experimental data for each isotherm was fitted by minimization of the SSE, described by Equation (3). Q_e,i_ is the experimental value, Q_f,i_ is the simulated value according to the isotherm model (*cf.* Equation 2), and N is the number of experimentally obtained data points.

(2) $$Q_{e}=\frac{Q_{m}{\left(K_{s}C_{e}\right)}^{n_{s}}}{1+{\left(K_{s}C_{e}\right)}^{n_{s}}}$$

(3) $$SSE=\sum \sqrt{\frac{{\left(Q_{e,i}-Q_{f,i}\right)}^{2}}{N}}$$

The dye-based sorption method [36] provides an independent estimate of the sorbent surface area (SA; m^2^/g) according to Equation 4.

(4) $$SA=\frac{A_{m}Q_{m}L}{Y}$$

where A*_m_* represents the cross-sectional area occupied by PNP (A*_m_* for a “*co-planar*” orientation is 5.25 × 10^−19^ m^2^/mol; whereas, an “*orthogonal*” orientation is 2.5 × 10^−19^ m^2^/mol), Q_m_ is the monolayer adsorption capacity per unit mass of sorbent, L is Avogadro’s number (mol^−1^), and Y is the coverage factor (Y = 1) for PNP [36].

## 3. Results and Discussion

β-CD copolymer materials were prepared at 1:3 β-CD:diisocyanate ratios where three mole equivalents of diisocyanate were reacted per mole of β-CD. The MDI-3 and CDI-3 copolymers were prepared at these monomer ratios to ensure the water insolubility of the copolymers and to compare their sorption properties with GAC. The structural characterization (e.g., IR, NMR, TGA, and porosimetry) of the copolymer materials containing β-CD are in good agreement with previously reported results and strongly support the identity of the sorbent materials used in this study [13,27,34].

**Figure 1:**
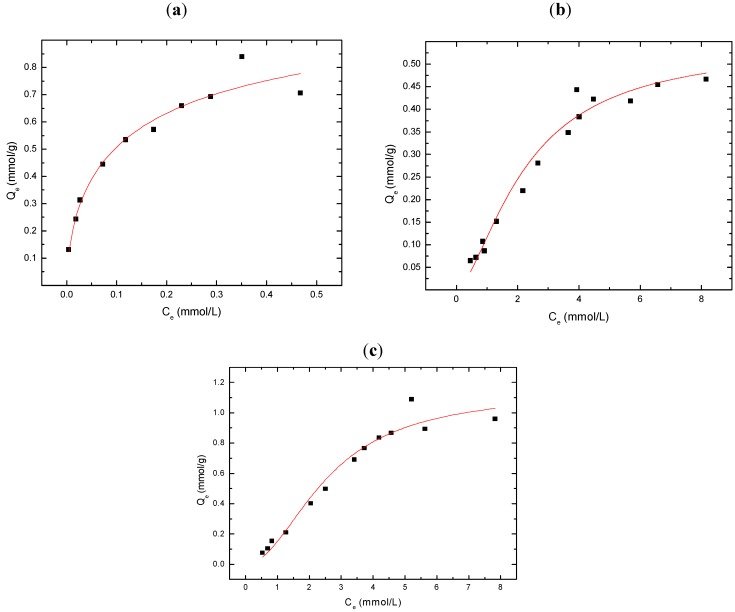
Sorption isotherms for 2,4-DCP with various sorbents at pH 9.0 and 295 K; (**a**) GAC; and (**b**) MDI-3, and (**c**) CDI-3. The solid line represents the best-fit curve according to the Sips isotherm (Equation 2).

Figure 1a-c illustrates typical sorption behavior (Q_e_ *vs*. C_e_) for GAC, CDI-3, and MDI-3 with 2,4-DCP at pH 9.0 in aqueous solution. Alkaline pH conditions were chosen throughout this study to ensure adequate solubility of the adsorbates (*cf*. Scheme 1) and to enable UV-Vis spectroscopic detection of the residual levels of adsorbate for the sorption analyses. At these experimental conditions, 2,4-DCP is fully ionized (pK_a_ = 7.8) and exists as the phenolate anion form. In general, the value of Q_e_ increases monotonically as C_e_ increases for each of the various materials. GAC and CDI-3 display the greatest overall sorption capacity (Q_m_) and reach saturation with 2,4-DCP at relatively low C_e_ values (~0.5 mM and 8 mM, respectively). In contrast, MDI-3 displays reduced sorption but plateaus ~8 mM, similar to that observed for CDI-3. The solid lines through the experimental data in Figure 1 represent the best-fit according to the Sips isotherm (Equation 3). The corresponding best-fit parameters are provided in Table 1 for GAC, MDI-3, and CDI-3 with 2,4-DCP. The relative ordering of the sorption capacity (Q_m_) for the sorbents is as follows: GAC ≈ CDI-3 > MDI-3. The adsorbate-sorbent binding affinity (K_S_) for 2,4-DCP is as follows: CDI-3 > MDI-3 > GAC. The relatively high Q_m_ value for GAC is attributed to its large BET surface area GAC (~10^3^ m^2^/g); whereas, the BET surface area estimates of the urethane copolymers are typically much lower (~10^1^ m^2^/g). A comparable Q_m_ value for CDI-3 and GAC is attributed to the relative magnitude of K_S_ and the occurrence of swelling in aqueous solution for the copolymer. Swelling phenomena of “*soft materials*” such as urethane copolymers have been described previously [34] and contribute to far greater sorbent surface areas (SA) in their hydrated state, as compared with BET estimates, derived according to porosimetry.

In contrast, GAC is regarded as a “*rigid material*” that does not undergo any substantial swelling in its dry state *vs.* the hydrated state. Therefore, the SA estimates are provided in Table 2 for the copolymer materials along with the dye-based estimates according to Equation 4 [36]. The sorption capacity of the urethane copolymers and GAC are unique in relation to other sorption studies because of the higher adsorbate concentrations employed herein for the sorption of the chlorinated compounds [31,32] compared with chromatographic studies. It is important to note that adsorbate concentrations at higher levels (*i.e.*, mM range) will result in attenuated removal efficiencies (ε_R_; Equation 5) relative to adsorbate concentrations at trace levels (*i.e.* μM range), and this is readily understood in terms of the partition coefficient for a given amount of adsorbate.

(5) $$\varepsilon_{R}=\left(\frac{C_{o}-C_{e}}{C_{o}}\right)\times 100\%$$

C_o_ and C_e_ are defined as in Equation 1; where the removal efficiencies are dependent on the pH, temperature, concentration, and the relative amount of the copolymer material [11,37].

**Table 1:** Isotherm sorption parameters provided by the best-fit according to the Sips model at 295 K.

2,4 DCP		**GAC**	**MDI-3**	**CDI-3**
	Q_m_ (mmol/g)	1.17	0.542	1.14
	K_S_ (mM^−1^)	656	444	391
	n_s_	0.619	1.59	2.00
2,4 D	Q_m_ (mmol/g)	0.641	0.607	0.0394
	K_S_ (mM^−1^)	1.28 × 10^3^	929	1.56
	n_s_	0.390	2.08	1.08
PCP	Q_m_ (mmol/g)	0.306	0.0860	0.0540
	K_S_ (mM^−1^)	51.2	2.17	24.0
	n_s_	0.816	0.717	1.65

**Table 2:** Textural parameters of nanoporous copolymer materials derived from nitrogen adsorption at 77 K and dye-based estimates of the surface area (SA), V_ads_, and Q_m_ for the copolymer materials at 295 K and pH 4.60 in aqueous solutions containing PNP.

Sorbent Material	SA (BET) (m^2^/g) ^a^	Dye Method SA (m^2^/g) ^b^	V_ads_(m^3^/g) ^c^	Q_m_(mmol/g) ^d^
CDI-3	0.332	620	33.9	1.96
MDI-3	2.96	449	24.6	1.42
GAC	951	641	35.1	35.1

^a^ BET Surface area, as determined from multi-point BET analysis; ^b^ Dye-based SA estimate using PNP at pH 4.6 in aqueous solution (Equation 4); ^c^ V_ads_ (m^3^/g) as in Equation (4) where Y = 1 and V_PNP_ = 0.64 nm × 0.43 nm × 0.33 nm = 9.08 × 10^−26^ m^3^; ^d^ Q_m_ for PNP according to the Sips isotherm (equation 2); The SA (BET) for crystalline β-CD = 0.635 m^2^/g.

**Figure 2:**
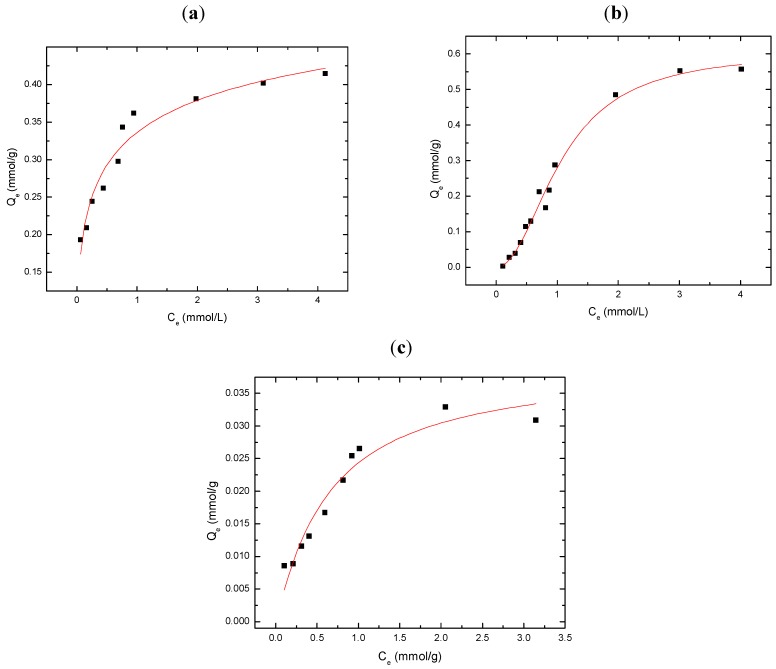
Sorption isotherms for 2,4-D with various sorbents at pH 9.0 and 295 K; (**a**) GAC, (**b**) MDI-3, and (**c**) CDI-3. The solid line represents the best-fit curve according to the Sips isotherm (Equation 2).

Figure 2a-c illustrates the sorption behavior (Q_e_ *vs*. C_e_) for GAC, CDI-3, and MDI-3 with 2,4-D at pH 9.0 in aqueous solution. At these experimental conditions, 2,4-D is fully ionized (pK_a_ = 2.6–3.3) and exists as the carboxylate anion. In all cases, the sorption increases monotonically with concentration in a manner similar to the results presented in Figure 1a-c. The relative ordering of the sorption capacity (Q_m_) for the sorbents with 2,4-D is as follows: GAC ≈ MDI-3 > CDI-3. At low values of C_e_, MDI-3 display greater sorption relative to GAC, its sorption capacity (Q_m_) reaches saturation ~3 mM with 2,4-D; whereas, GAC reaches a plateau ~4 mM. In contrast, CDI-3 displays reduced sorption and approaches saturation beyond 3.5 mM, comparable to that observed for GAC. The corresponding parameters for the Sips isotherm are provided in Table 1 for these sorbents with 2,4-D.

The relative binding affinity (K_S_) between the sorbents and 2,4-D is as follows: GAC > CDI-3 > MDI-3. The main difference between 2,4-D and 2,4-DCP is the nature of the anion. In the former case, the anion charge is located on the carboxylate ion and does not reside on the aromatic ring. In contrast, the anion charge for 2,4-D is delocalized over the arene ring system for such phenolate anions. The difference in electron density of these two species contributes to the differing binding affinity to the inclusion sites and the linker domains of the copolymer materials. In the case of MDI-3, the occurrence of favorable π–π interactions are anticipated for 2,4-D relative to 2,4-DCP because of the aromatic crosslinker domains of the copolymer. π–π interactions were similarly concluded for 2,4-D and GAC [38,39]. In comparison with GAC, the slightly lower SA values of the urethane copolymers are offset by the occurrence of favorable inclusion at the β-CD sites of the copolymer [27]. The Q_m_ value in Table 1 for MDI-3 (0.542 mmol/g) exceeds the value reported a β-CD-epichlorohydrin copolymer with 2,4-DCP (Q_m_ = 0.16 mmol/g) [40]. The involvement of inclusion binding at the β-CD sites of the urethane copolymer is further supported by independent sorption studies of different β-CD-based copolymer materials [41,42,43]. According to Table 1, the Q_m_ value for MDI-3 and 2,4-D is 134 mg/g, and is greater than a published value for a polypropylene nonwoven fabric containing α-CD for (Q_m_ = 41.1 mg/g) [44]. Notwithstanding the differences in binding affinity between α-CD and β-CD with 2,4-D, the foregoing results qualitatively support that the urethane copolymers described herein have favorable inclusion site accessibility.

**Figure 3:**
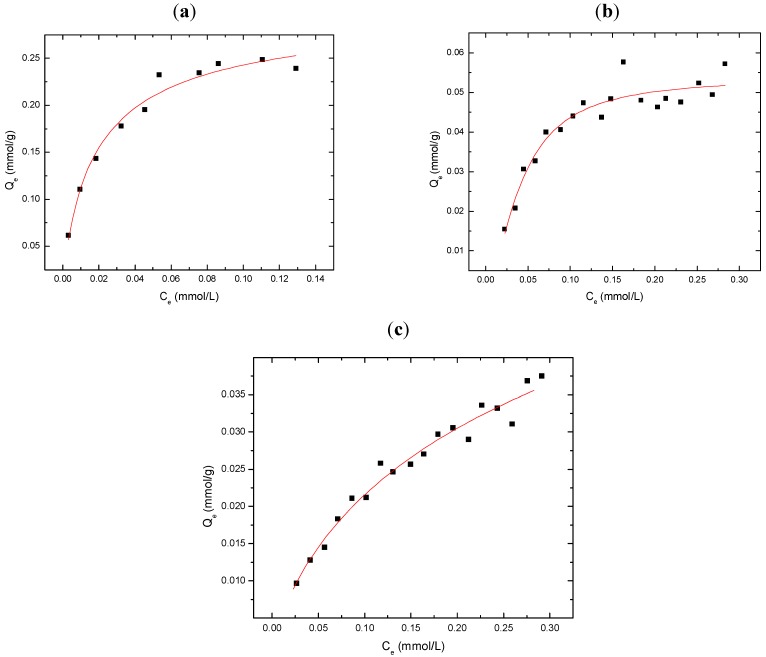
Sorption isotherms for PCP with various sorbents at pH 9.0 and 295 K; (**a**) GAC; (**b**) CDI-3; and (**c**) MDI-3. The solid line represents the best-fit curve according to the Sips isotherm (Equation 2).

Figure 3a-c illustrates the sorption behavior (Q_e_ *vs*. C_e_) for GAC, CDI-3, and MDI-3 with PCP at pH 9 in aqueous solution. At these experimental conditions, PCP is fully ionized (pK_a_ = 4.74) and exists as the phenoxide anion. In all cases, the sorption increases monotonically with concentration in a manner similar to the results presented in Figure 1; however, the overall sorption is significantly attenuated up to C_e_ ~0.3 mM. The reduced sorption for PCP is surprising in spite of its apolar characteristics and a previous report which concludes significant removal of PCP (*i.e.* ε_R_ ~ 99%) with urethane copolymer materials [31,32]. An HPLC study by Paleologou *et al.* reports that highly chlorinated congeners exhibit restricted inclusion binding to the stationary phase containing grafted CDs [45]. The restricted access to the inclusion sites may be related to steric effects in the annular hydroxyl region resulting from the copolymer framework structure, as detailed in a recent study of the inclusion site accessibility of related copolymer materials [27].

The relative sorption capacity (Q_m_) for each sorbent with PCP is as follows: GAC > MDI-3 ≈ CDI-3. At low values of C_e_, MDI-3 displays greater sorption relative to GAC, and its sorption capacity (Q_m_) is reached ~0.15 mM with PCP; whereas, GAC reaches saturation for C_e_ values > 0.3 mM. In contrast, CDI-3 displays comparable sorption properties to MDI-3 and levels off ~0.2 mM. The relative binding affinity (K_S_) between the sorbents and PCP is as follows: GAC > MDI-3 ≈ CDI-3. The attenuated binding affinity observed for the copolymers with PCP is attributed to steric effects in the annular region of β-CD arising from the urethane bonds of the copolymer framework [14]. In comparison with 2,4-DCP, pentachlorophenol (PCP) is a larger adsorbate and the attenuated sorption behavior observed for the copolymers may be related to the reduced inclusion site accessibility, as reported for these types of urethane copolymer sorbent materials at greater crosslinking ratios [27]. It is noteworthy to recognize that the van der Waals diameter of a –Cl atom is similar to that for a –CH_3_ group and provides insight regarding the observed steric effects for dichlorinated *vs.* polychlorinated phenols. The difference in electron density of 2,4-DCP and PCP may contribute to the differing binding affinity to the β-CD inclusion sites; however, improper *size-fit* matching of the host-guest complex is concluded to be the major factor for the attenuated inclusion of PCP by β-CD [45]. In the case of MDI-3, the occurrence of favorable π–π interactions and charge delocalization of the anion are anticipated to favour the sorption of PCP. Favorable π–π interactions and hydrophobic effects are also anticipated with GAC [39]. According to Table 1, the Q_m_ value for MDI-3/PCP is 23 mg/g and is lower than a reported value for a polypropylene nonwoven fabric containing grafted α-CD (Q_m_ = 75.2 mg/g) [44]. Notwithstanding the differences in binding affinity between α-CD and β-CD with PCP according to the size-fit matching of the host-guest complex, the foregoing results qualitatively support that the urethane copolymers described herein have attenuated inclusion site accessibility resulting from steric effects in the annular hydroxyl region of β-CD due to crosslinking [27], as illustrated in Scheme 2. In comparison with GAC, the lower SA values of the urethane copolymers are offset by the favorable interactions with the β-CD inclusion sites of the copolymer sorbent [46,47,48].

**Figure 4:**
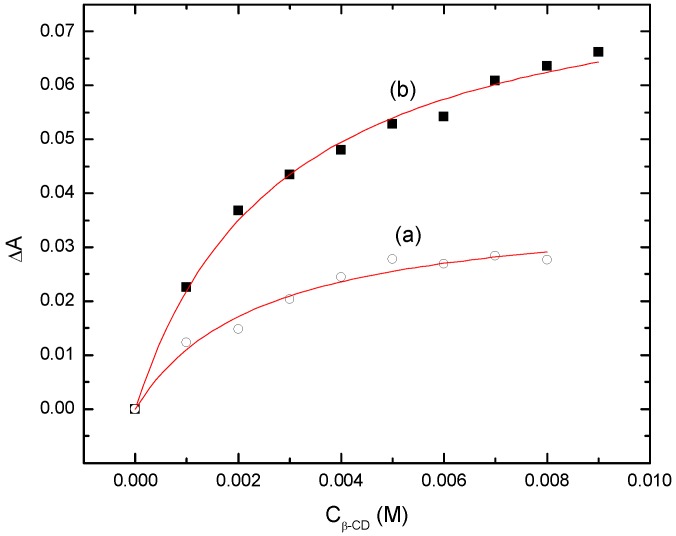
UV-Vis titration isotherms for β-CD and guest molecules at pH 9.0 and 295 K; (**a**) 2,4-D where λ_max_ = 290 nm; and (**b**) 2,4-DCP where λ_max_ = 305 nm. The solid lines represent the nonlinear least squares fit, according to a 1:1 equilibrium host-guest binding model [27].

Independent evidence of the formation of discrete 1:1 inclusion complexes is provided by isothermal UV-Vis, NMR, and calorimetric titrations for 2,4-D and 2,4-DCP with β-CD [49,50,51]. The 1:1 binding constants were estimated in aqueous solution at variable pH conditions relative to this sorption study (*cf*. Table 3). 1:1 binding constants have been reported for the 1:1 β-CD/PCP complexes (K = 258 L/mol at pH =7 and K = 309 L/mol at pH = 3) [52]. The values are comparable in magnitude with those for PNP at pH 4.6 and 9.0 provided in Table 3. The enhanced 1:1 binding affinity of PNP at alkaline pH (*i.e.* phenolate ion) relative to pH values below its pK_a_ (7.15) have been independently reported by Fan *et al.* [53] and may be related to polarizability and hydration effects [54] of the anion species. Thus, it can be concluded that stable complexes are formed between β-CD and chlorinated aromatic adsorbates in their non-ionized and ionized forms according to the results for β-CD in Figure 4 and Table 3. In the case of urethane copolymers containing β-CD, enhanced sorption of PNP is observed at pH ≤ 7.15 since the linker domains contribute to sorption in addition to the β-CD inclusion sites [14]. At pH values above the pK_a_ of the adsorbates examined herein, inclusion complexes are anticipated to dominate the sorption properties observed for urethane copolymer sorbents [14].

**Table 3:** Experimental and literature 1:1 binding constants for β-CD/guest complexes in aqueous solution at 295 K and various pH conditions according to spectroscopic and calorimetric titrations.

Adsorbate	K_eq_ (L.mol^−1^) pH = 4.6	K_eq_ (L.mol^−1^) pH = 9.0
**2,4-DCP**	245 (350 ± 23)^49^ (556 ± 17)^49^	350
**2,4-D**	197 (63.58 ± 5.77) ^50^	410
**PNP**	338 (159)^51^	370 (1549)^51^

**Note:** λ_max_ = 290 nm (2,4-D at pH 4.6 and 9.0); λ_max_ = 305 nm (2,4-DCP at pH 4.6 and 9.0); λ_max_ = 404 nm (PNP at pH 9.0); and λ_max_ = 317 nm (PNP at pH 4.6). The 1:1 binding constants in parentheses represent literature values.

## 4. Conclusions

Urethane-based sorbent materials that incorporate β-cyclodextrin exhibit favorable adsorption of chlorinated aromatic adsorbates in aqueous solutions at alkaline pH. Differences in the sorption properties were observed according to the surface area and chemical nature of the sorbents. The sorption properties are quantitatively supported by the Sips parameters and the textural properties of the materials, respectively. Overall, the sorption capacity was greatest for GAC; however, the binding affinity is more favorable for CD-based urethane copolymer materials in spite of their lower effective surface areas. Inclusion complexes are predominantly formed for the copolymer sorbents with PCP and 2,4-D adsorbates. Weakly bound complexes are formed in the case of PCP due to the size of this adsorbate and the accompanying steric effects in the annular hydroxyl region of β-CD arising from the copolymer framework. The variation in the sorption capacity of the various adsorbates with the copolymers is understood according to the *goodness-of-fit* between the host and guest along with the hydrophile-lipophile characteristics of the guest, resulting in the formation of inclusion and interfacial bound adsorbates (*cf.* Scheme 2). The results described herein outline the potential suitability and application of CD-based sorbents for use in aquatic/soil environments for the sequestration of low molecular weight organic aromatic compounds (e.g., dichlorinated and polychlorophenols). The tunable physicochemical properties of such crosslinked sorbent materials described herein are suitable for fluidized bed reactors [55,56] for the sequestration of biocides [54,55] in contaminated drinking water reservoirs. As well, copolymer sorbents containing β-CD may provide improved technologies for biocide optimization and usage; thereby minimizing financial losses, health hazards, and environmental contamination [54,55].

## References

[1] Sun Y., Chen J.L., Li A.M., Liu F.Q., Zhang Q.X. (2005). Adsorption of resorcinol and catechol from aqueous solution by aminated hypercrosslinked polymers. React. Funct. Polym..

[2] Yin J., Chen R., Ji Y., Zhao C., Zhao G., Zhang H. (2010). Adsorption of phenols by magnetic polysulfone microcapsules containing tributyl phosphate. Chem. Eng. J..

[3] Onodera S. (2010). Formation mechanism and chemical safety of nonintentional chemical substances present in chlorinated drinking water and wastewater. Yakugaku Zasshi.

[4] Kawakami T., Nishi I., Kishi T., Onodera S. (2008). Formation of polybrominated and polychlorinated alkylphenoxyalkylphenols (PXAPAPs) during aqueous chlorination of 4-alkylphenol solutions in the presence of bromide ion. Organohalogen Compd..

[5] Macdonald R.W., Ikonomou M.G., Paton D.W. (1998). Historical inputs of PCDDs, PCDFs, and PCBs to a British Columbia interior lake: The effect of environmental controls on pulp mill emissions. Environ. Sci. Technol..

[6] Onodera S., Ogawa M., Yamawaki C., Yamagishi K., Suzuki S. (1989). Production of polychlorinated phenoxyphenols (predioxins) by aqueous chlorination of organic compounds. Chemosphere.

[7] Nowak K.M., Miltner A., Gehre M., Schaeffer A., Kaestner M. (2011). Formation and fate of bound residues from microbial biomass during 2,4-D degradation in soil. Environ. Sci. Technol..

[8] Schwartz F.W., Lee E.S., Kim Y. (2008). Lessons from practice in the assessment and remediation of contaminated ground water. Geosci. J..

[9] End Poverty 2015: Millennium development goals. http://www.un.org/millenniumgoals/.

[10] Wenz G. (1994). Cyclodextrins as building blocks for supramolecular structures and functional units. Angew. Chem. Int. Ed. Engl..

[11] Crini G., Bertini S., Torri G., Naggi A., Sforzini D., Vecchi C., Janus L., Lekchiri Y., Morcellet M. (1998). Sorption of aromatic compounds in water using insoluble cyclodextrin polymers. J. Appl. Polym. Sci..

[12] Harada A., Hashidzume A., Takashima Y., Abe A., Albertsson A.-C., Duncan R., Dušek K., de Jeu W.H., Joanny J.-F., Kausch H.-H., Kobayashi S., Lee K.-S., Leibler L. (2006). Supramolecular Polymers, Polymeric Betains, Oligomers (Advances in Polymer Science).

[13] Mohamed M.H., Wilson L.D., Headley J.V., Peru K.M. (2008). Novel materials for environmental remediation of tailing pond waters containing naphthenic acids. Process Saf. Environ. Prot..

[14] Mohamed M.H., Wilson L.D., Headley J.V., Peru K.M. (2011). Investigation of the sorption properties of β-Cyclodextrin based polyurethanes with phenolic dyes and naphthenates. J. Colloid Interface Sci..

[15] Crini G. (2005). Recent developments in polysaccharide-based materials used as adsorbents in wastewater treatment. Prog. Polym. Sci..

[16] van de Manakker F., Vermonden T., van Nostrum C.F., Hennink W.E. (2009). Cyclodextrin-based polymeric materials: Synthesis, properties, and pharmaceutical/biomedical applications. Biomacromolecules.

[17] Orprecio R., Evans C.H. (2003). Polymer-immobilized cyclodextrin trapping of model organic pollutants in flowing water streams. J. Appl. Polym. Sci..

[18] Janus L., Crini G., El-Rezzi V., Morcellet M., Cambiaghi A., Torri G., Naggi A., Vecchi C. (1999). New sorbents containing β-cyclodextrin. Synthesis, characterization, and sorption properties. React. Funct. Polym..

[19] Topchieva I.N., Kalashnikov F.A., Spiridonov V.V., Mel’nikov A.B., Polushina G.E., Lezov A.V. (2003). Cyclodextrin-containing nanotubes as a base for designing materials with a new architecture. Dokl. Chem..

[20] Wintgens V., Amiel C. (2005). Surface plasmon resonance study of the interaction of a beta-cyclodextrin polymer and hydrophobically modified poly(N-isopropylacrylamide). Langmuir..

[21] Vélaz I., Isasi J., Sánchez M., Uzqueda M., Ponchel G. (2007). Structural characteristics of some soluble and insoluble β-cyclodextrin polymers. J. Incl. Phenom. Macrocycl. Chem..

[22] Bender M.L., Komiyama M. (1978). Cyclodextrin Chemistry.

[23] Inoue Y., Harkushi T., Liu Y., Tong L.H., Shen B.J., Jin D.S. (1993). Thermodynamics of molecular recognition by cyclodextrins. 1. Calorimetric titration of inclusion complexation of naphthalenesulfonates with alpha-, beta-, and gamma-cyclodextrins: Enthalpy-entropy compensation. J. Am. Chem. Soc..

[24] Polarz S., Antonietti M. (2002). Porous materials *via* nanocasting procedures: Innovative materials and learning about soft-matter organization. Chem. Commun..

[25] Asouhidou D.D., Triantafyllidis K.S., Lazaridis N.K., Matis K.A. (2009). Adsorption of Remazol Red 3BS from aqueous solutions using APTES-and cyclodextrin-modified HMS-type mesoporous silicas. Colloids Surf. A.

[26] Shiu W., Ma K., Varhanickova D., Mackay D. (1994). Chlorophenols and alkylphenols: A review and correlation of environmentally relevant properties and fate in an evaluative environment. Chemosphere.

[27] Mohamed M.H., Wilson L.D., Headley J.V. (2010). Determination of host-guest binding sites for β-cyclodextrin urethane copolymers. Carbohydr. Polym..

[28] Kleine R., Hamera P. (1997). Use of immobilized β-cyclodextrin for decontamination of water. CLB Chem. Labor und Biotech..

[29] Bibby A., Mercier L. (2003). Adsorption and separation of water-soluble aromatic molecules by cyclodextrin-functionalized meosporous silica. Green Chem..

[30] Ma M., Li D. (1999). New organic nanoporous polymers and their inclusion complexes. Chem. Mat..

[31] Mamba B.B., Krause R.W., Malefetse T.J., Nxumalo E.N. (2007). Monofunctionalized cyclodextrin polymers for the removal of organic pollutants from water. Environ. Chem. Lett..

[32] Mhlanga S.D., Mamba B.B., Krause R.W., Malefetse T.J. (2007). Removal of organic contaminants from water using nanosponge cyclodextrin polyurethanes. J. Chem. Tech. Biotech..

[33] Mohamed M.H., Wilson L.D., Headley J.V. (2011). Design and characterization of novel β-cyclodextrin based copolymer materials. Carbohydr. Res..

[34] Wilson L.D., Mohamed M.H., Headley J.V. (2011). Surface area and pore structure properties of β-cyclodextrin-urethane copolymer materials. J. Colloid Interface Sci..

[35] Sips R. (1948). On the Structure of a Catalyst Surface. J. Chem. Phys..

[36] Giles C.H., D’Silva A.P., Tridevi A.S. (1970). Use of *p*-nitrophenol for specific surface measurement of granular solids and fibres. J. Appl. Chem..

[37] Pratt D.Y., Wilson L.D., Kozinski J.A., Morhart A.M. (2010). Preparation and sorption studies of β-cyclodextrin/epicholohydrin copolymers. J. Appl. Polym. Sci..

[38] Pan J., Zou X., Wang X., Guan W., Li C., Yan Y., Wu X. (2011). Adsorptive removal of 2,4-dichlorophenol and 2,6-dichlorophenol from aqueous solution by β-cyclodextrin/attapulgite composites: Equilibrium, kinetics and thermodynamics. Chem. Eng. J..

[39] Steed J.W., Atwood J.L. (2009). Supramolecular Chemistry.

[40] Li N., Mei Z., Ding S. (2010). 2,4-Dichlorophenol sorption on cyclodextrin polymers. J. Incl. Phenom.Macrocycl. Chem..

[41] Crini G., Janus L., Morcellet M., Torri G., Naggi A., Bertini S., Vecchi C. (1998). Macroporous polyamines containing cyclodextrin: Synthesis, characterization, and sorption prperties. J. Appl. Polym. Sci..

[42] Carbonnier B., Janus L., Lekchiri Y., Morcellet M. (2004). Coating of porous silica beads by *in situ* polymerization/crosslinking of 2-hydroxypropyl β- cyclodextrin for reversed-phase high performance liquid chromatography applications. J. Appl. Polym. Sci..

[43] Martel B., Le Thuaut P., Bertini S., Crini G., Bacquet M., Torri G., Morcellet M. (2002). Grafting of cyclodextrins onto polypropylene nonwoven fabrics for the manufacture of reactive filters. III. Study of the sorption properties. J. Appl. Polym. Sci..

[44] Paleologou M., Li S., Purdy W.C. (1990). Liquid chromatographic retention behavior and separation of chlorophenols on a β-cyclodextrin bonded-phase column, part I. Monoaromatic chlorophenols: Retention behavior. J. Chromatogr. Sci..

[45] Hanna K., de Brauer C., Germain P. (2004). Cyclodextrin -enhanced solubilization of pentachlorophenol in water. J. Environ. Manag..

[46] Wang X., Brusseau M.L. (1995). Simultaneous complexation of organic compounds and heavy metals by a modified cyclodextrin. Environ. Sci. Technol..

[47] Phan T.N.T., Bacquet M., Morcellet M. (2002). The removal of organic pollutants from water using new silica-supported β-cyclodextrin derivatives. React. Funct. Polym..

[48] Fenyvesi E., Szeman J., Szejtli J. (1996). Extraction of PAHs and pesticides from contaminated soils with aqueous CD solutions. J. Inclusion Phenom. Mol. Recognit. Chem..

[49] Leyva E., Moctezuma E., Strouse J., Garcia-Garibay M.A. (2001). Spectrometric and 2D NMR studies on the complexation of chlorophenols with cyclodextrins. J. Incl. Phenom. Macrocycl. Chem..

[50] Almansa-Lopez E., Bosque-Sendra J.M., Cuadros-Rodriguez L., Garcia-Campana A.M., Aaron J.J. (2003). Applying non-parametric statistical methods to the classical measurements of inclusion complex binding constants. Anal. Bioanal.Chem..

[51] Eftink M.R., Harrison J.C. (1981). Calorimetric Studies of p-Nitrophenol Binding to α- and β-Cyclodextrin. Bioorg. Chem..

[52] Fan Y., Feng Y.-Q., Da S.-L., Feng P.-Y. (2003). Evaluation of β-cyclodextrin bonded silica as a selective sorbent for the solid-phase extraction of 4-nitrophenol and 2,4-dinitrophenol. Anal. Sci..

[53] Gerasimowicz W., Wojcik J. (1982). Azo dye-α-cyclodextrin adduct formation. Bioorg. Chem..

[54] Torrents A., Jayasundera S. Effects of sorbent properties on sorption of agrochemical. Proceedings of the 211th ACS National Meeting.

[55] Sevillano X., Isasi J.R., Penas F.J. (2008). Feasibility study of degradation of phenol in a fluidized bed bioreactor with a cyclodextrin polymer as biofilm carrier. Biodegradation.

[56] Tardioli P.W., Zanin G.M., de Moraes F.F. (2000). Production of cyclodextrins in a fluidized-bed reactor using cyclodextrin -glycosyl-transferase. Appl. Biochem. Biotech..

